# Adaptive Autoregressive Model for Reduction of Noise in SPECT

**DOI:** 10.1155/2015/494691

**Published:** 2015-05-18

**Authors:** Reijo Takalo, Heli Hytti, Heimo Ihalainen, Antti Sohlberg

**Affiliations:** ^1^Division of Nuclear Medicine, Department of Diagnostic Radiology, Oulu University Hospital (OYS), P.O. Box 500, 90029 Oulu, Finland; ^2^Department of Automation Science and Engineering, Tampere University of Technology, P.O. Box 692, 33101 Tampere, Finland; ^3^Joint Authority for Päijät-Häme Social and Health Care, Department of Clinical Physiology and Nuclear Medicine, Keskussairaalankatu 7, 15850 Lahti, Finland

## Abstract

This paper presents improved autoregressive modelling (AR) to reduce noise in SPECT images. An AR filter was applied to prefilter projection images and postfilter ordered subset expectation maximisation (OSEM) reconstruction images (AR-OSEM-AR method). The performance of this method was compared with filtered back projection (FBP) preceded by Butterworth filtering (BW-FBP method) and the OSEM reconstruction method followed by Butterworth filtering (OSEM-BW method). A mathematical cylinder phantom was used for the study. It consisted of hot and cold objects. The tests were performed using three simulated SPECT datasets. Image quality was assessed by means of the percentage contrast resolution (CR%) and the full width at half maximum (FWHM) of the line spread functions of the cylinders. The BW-FBP method showed the highest CR% values and the AR-OSEM-AR method gave the lowest CR% values for cold stacks. In the analysis of hot stacks, the BW-FBP method had higher CR% values than the OSEM-BW method. The BW-FBP method exhibited the lowest FWHM values for cold stacks and the AR-OSEM-AR method for hot stacks. In conclusion, the AR-OSEM-AR method is a feasible way to remove noise from SPECT images. It has good spatial resolution for hot objects.

## 1. Introduction

Numerous methods for removing noise from SPECT images have been proposed [1, 2]. This indicates the difficulty of the task. Noise removal can be performed before reconstruction (prefiltering), during reconstructions or after reconstruction (postfiltering). In modern iterative methods, collimator correction denoises images during reconstruction, but the reconstructed images may still require postfiltering [3]. Earlier we introduced an adaptive autoregressive (AR) filter to reduce noise in scintigraphic planar images or projection images of a SPECT study [4]. In the present work, the AR filter was further improved to reduce noise from the projection images and also from three-dimensionally reconstructed data. It is important to apply the best AR filter to the projection data of SPECT, because a small change in the projection data may cause a large change in the estimated transaxial image [5]. Our method was compared with two established methods for improving image quality in SPECT. The methodical comparison was carried out using a three-dimensional mathematical cylinder phantom (3D-MAC) [6], and it was illustrated with patient data.

## 2. Methods

### 2.1. AR Model

In two-dimensional AR modelling, each value of an image is regressed on its neighbourhood pixel values, called the prediction region. An AR model can be regarded as a low-pass filter that divides the image into two additive components, a predictable image and a prediction error image. An AR process *X*(*n*_1_, *n*_2_) is defined by

(1) Xpredn1,n2 =−∑k1∑k2ak1,k2Xorign1−k1,n2−k2+wn1,n2,

where *a*(*k*_1_, *k*_2_) are the predictor (weighting) coefficients, indices *k*_1_ and *k*_2_ define the type of prediction region in a two-dimensional array (*n*_1_, *n*_2_ matrix), and *w*(*n*_1_, *n*_2_) represents prediction error, that is, the difference between the predicted value and the current value in this pixel. The predictable image *X*_pred_ is the image obtained by applying the AR model to the original image *X*_orig_. The prediction error image *X*_*err*⁡_ = *X*_orig_ − *X*_pred_.

**Figure 1:**
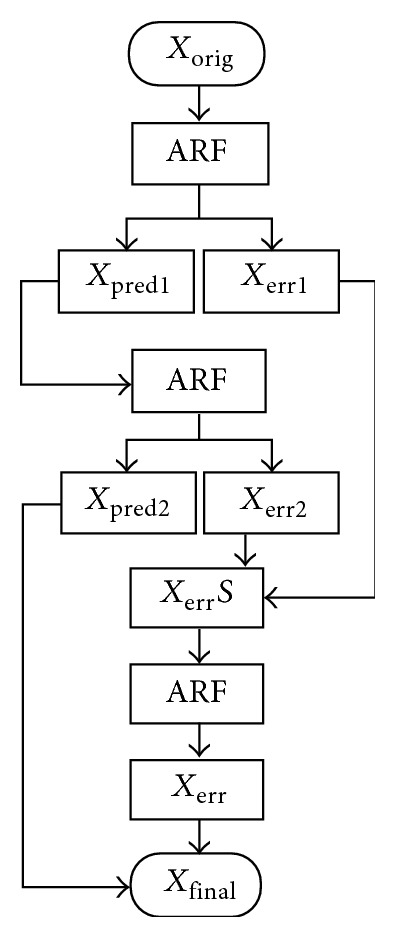
Flowchart of the autoregressive denoising process. ARF: autoregressive filtering; *X*_orig_: original noise-corrupted image; *X*_pred1_ and *X*_pred2_: predictable images; *X*_*err*⁡1_, *X*_*err*⁡2_, *X*_*err*⁡_: prediction error images;  *X*_*err*⁡_ *S*: sum of two prediction error images; *X*_final_: final image.

In a typical scintigraphic image, there are large local spatial variations in the count number of the image. Therefore, the same model cannot be applied to the entire image, but the model must be adapted to the variations. In this adaptive method, the image area is divided into smaller blocks and the AR model is then fitted into each block separately by using MATLAB subroutines. Recently, a block-wise denoising method has been introduced also for three-dimensional ultrasound images [7]. In the AR model, a prediction region of four orthogonal neighbours of the predicted pixel with a block size of 5 × 5 pixels was used [4]. Seventy-five percent overlap of the image blocks in combination with one iteration of the filtering procedure was used. The two error term images were summed up and subjected to AR filtering, and the resulting image was then added to the iteratively filtered image (Figure 1). In the present study, we tested the effect of using another AR model for the summed error term images than for the original image. We used the same transaxial slice of the Zubal phantom [8] and the same simulation conditions as in our previous work [4], and image quality was assessed by means of the mean squared error (MSE) of the image. It is of note that the Poisson-noise-corrupted slice of the phantom actually represented an artificial scintigraphic planar image or a projection image of a SPECT study. The AR model with the lowest MSE was then used to prefilter the SPECT projection images and also to postfilter iteratively reconstructed data. The filter was applied to each set of orthogonal plane images separately. The software was based on MATLAB subroutines (The MathWorks, Inc.).

### 2.2. Phantom

Data were simulated using a 3D-MAC phantom [6]. The phantom measured 200 mm in both diameter and length. It comprised three imbedded objects: two hot objects and a cold one. Each object consisted of five stacked cylinders. The cylinders had diameters of 4, 10, 20, 40, and 60 mm and a length of 30 mm. The smallest cylinder was not utilised in the present study because its dimensions were beyond the resolution of the simulated SPECT system used. Relative activities were 1, 0, 2, and 4 for the background, a cold stack, and two hot stacks, respectively. The tests were performed using three SPECT datasets with different image statistics. Total counts of the projection images were approximately 50000 (low level), 100000 (intermediate level), and 150000 (high level) per projection. A built-in MATLAB function was used to add Poisson noise to ideal projection images of the 3D-MAC. The mean counts of a pixel in the projection images were 12, 24, and 37, respectively, and the range of pixel values was 0–52, 0–104, and 0–156, respectively. The matrix size was 64 × 64 pixels, pixel size was 4 mm, and the number of projections was 120. There was no scatter or attenuation component and perfect depth-independent resolution was assumed in the simulated data. Thus, the only factor degrading image quality in the projection images was the Poisson noise.

### 2.3. Reconstruction Methods

Transaxial slices were reconstructed using either the filtered back projection method (FBP) [9] or an iterative ordered subset expectation maximisation (OSEM) algorithm [10]. The reconstruction methods were implemented on the Hermes SPECT (G) reconstruction software (version 3.8) and reconstruction engine of Hermes HybridRecon (Hermes Medical Solutions, Stockholm, Sweden), respectively. Three methods were compared: AR filtering before and after ordered OSEM reconstruction (AR-OSEM-AR), two-dimensional Butterworth filtering before FBP reconstruction in combination with a ramp filter during reconstruction (BW-FBP), and OSEM reconstruction followed by three-dimensional Butterworth filtering (OSEM-BW). The Butterworth filter was originally designed for one-dimensional data [11]. In the OSEM method, the number of subsets was set to 8 and the number of iterations to 10. Postfiltering was performed using Multimodality software (Hermes Medical Solutions, Stockholm, Sweden). Noise-free projection images were also reconstructed using the OSEM (Ideal-OSEM) method.

### 2.4. Assessment of Image Quality

To obtain a fair comparison of the methods, the same amount of filtering was applied in each method. This was done by drawing a circular region-of-interest (ROI) 150 mm in diameter in the uniform part of the phantom and calculating the percentage coefficient of variation (CoV%) in the ROI, that is, the ratio of the standard deviation to the mean multiplied by 100. This kind of presentation ensures that filtering between each method is equal.

Percentage contrast resolution (CR%) values for the activity in each cylinder and uniform activity were calculated. CR% can be expressed by the following formula [12]:

(2) $$\begin{array}{c} \text{CR}\%=\frac{\left|M-m\right|}{M}\times 100\%, \end{array}$$

where *M* is the count value of uniform activity and *m* is the count value in each cylinder. Activity in each cylinder was analysed using a circular ROI with the same diameter as the cylinder. The ROIs were drawn on noise-free transaxial slices and were copied to each set of reconstructed data, so their position and area were equal in every image. The ROIs were drawn using Multimodality software. The CR% values were obtained using the average counts in the ROIs.

Spatial resolution was estimated by the full width at half maximum (FWHM) of the line spread functions of the cylinders. One-, two-, four- and six-pixel-thick profiles were drawn through the 10-, 20-, 40- and 60-mm-wide cylinders, respectively. The FWMH values were calculated using Hermes quality control software (version 2.0).

### 2.5. Patient Study

Skeletal SPECT was performed three hours after an intravenous injection of 925 MBq of ^99m^Technetium-labelled methylene diphosphonate. The images were obtained over a 360° arc, using 64 projections at 20 sec per projection. The images were acquired into a 128 × 128 matrix with a pixel size of 4.8 mm. Total counts of the projection images were 41006–66830 counts per projection.

### 2.6. Statistical Methods

The data were analysed using WinSTAT for Excel (version 2007.1; R. Fitch Software, Staufen, Germany). Pair-wise comparisons were performed with the nonparametric Wilcoxon's rank-sum test. Comparisons were made between the AR-OSEM-AR and BW-FBP methods, the AR-OSEM-AR and OSEM-BW methods, and the BW-FBP and OSEM-BW methods. Data from the cold stacks and the pooled hot stacks were analysed separately. For each cylinder, the paired difference between the values of a variable was computed. The values of the differences were sorted to get a rank order. Finally, the mean rank of negative differences was compared with that of positive differences. Wilcoxon's rank-sum test determines to what extent the difference in mean rank is significant. A *P* value of less than 0.05 was considered significant.

## 3. Results

**Figure 2:**
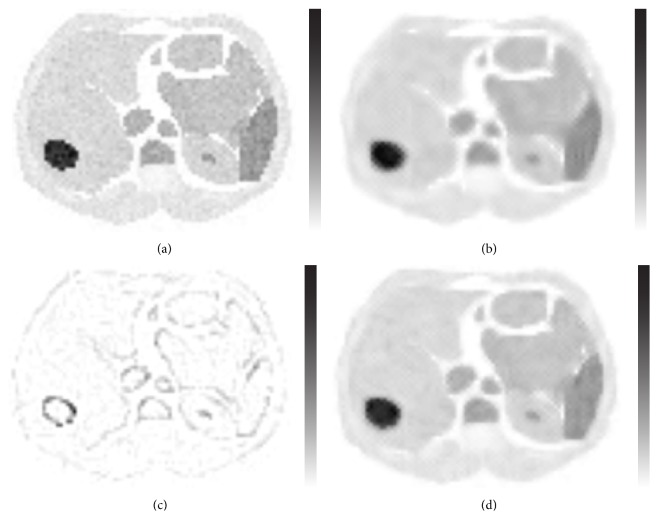
Transaxial slice of the Zubal phantom. (a) Poisson-noise-corrupted transaxial slice. (b) Iteratively filtered predictable image. (c) Filtered summed error term image. (d) The final image. The images are individually scaled to their own maximum. Inverse linear grey scale is used for comparison with original phantom. The total count level is 108791 in the Poisson-noise-corrupted image, 102237 in the iteratively filtered predictable image, and 6205 in the filtered summed error term image.

The MSE of the images improved when a different AR model was used for the summed error term image rather than for the original image. A prediction region of four orthogonal neighbours with a block size of 5 × 5 pixels for the original image and a prediction region of 3 × 3 and a block size of 6 × 6 for the summed error term image produced the lowest MSE, although the differences were small (Table 1). Part of the counts at the edges of the image could be returned to the filtered image to reduce blurring of the image (Figure 2).

Butterworth filtering was chosen so that the methods had the same amount of statistical fluctuation in the uniform part of the phantom, as confirmed by the CoV% values (Table 2). For the cold stacks, the BW-FBP method showed higher CR% values than the AR-OSEM-AR and OSEM-BW methods (Table 3). The *P* values were 0.003 and 0.04, respectively. The BW-FBP method had the highest CR% values for all other cold cylinders except the two smallest cylinders at the intermediate count level. The OSEM-BW method, in turn, displayed better performance than the AR-OSEM-AR method (*P* = 0.002). When the hot stacks were assessed, there were no statistically significant differences between the AR-OSEM-AR and BW-FBP methods nor between the AR-OSEM-AR and OSEM-BW methods, but the BW-FBP method showed higher CR% values than the OSEM-BW method (*P* = 0.001).

In the analysis of spatial resolution, without exception, the BW-FBP and OSEM-BW methods exhibited lower FWHM values for the cold stacks than the AR-OSEM-AR method (*P* = 0.002 for both comparisons), but there was no statistical difference between the BW-FBP and OSEM-BW methods (Table 4). For the hot stacks, the AR-OSEM-AR method showed lower FWHM values than the BW-FBP and OSEM-BW methods. The *P* values were 0.01 and 0.04, respectively.

Furthermore, the OSEM-BW method had lower FWHM values than the BW-FBP method (*P* = 0.008). It is of note that the AR-OSEM-AR method showed better resolution than the other two methods in the analysis of the two smallest hot stacks, with two exceptions in the analysis of cylinders with two times the background activity and a diameter of 10 mm (Table 4).

**Figure 3:**
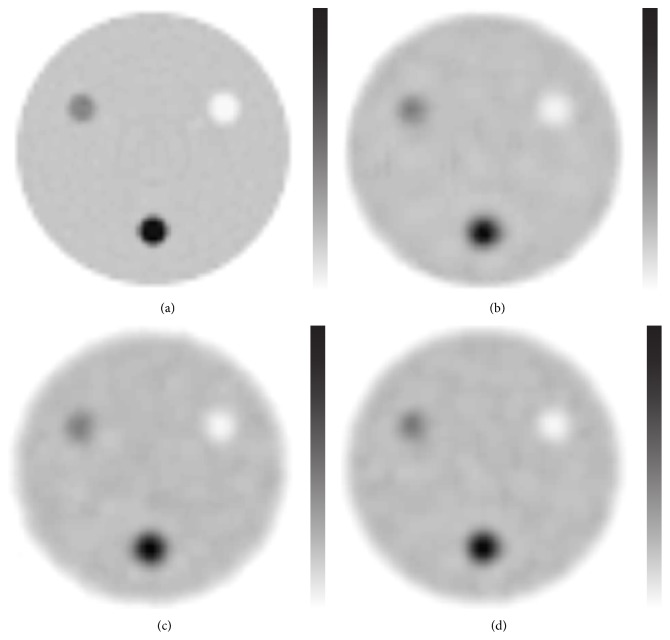
Transaxial slices of the phantom at the level of cylinders with a diameter of 20 mm. (a) Images reconstructed from noise-free projection images using ordered subset expectation maximisation reconstruction. (b) Autoregressive filtering before and after ordered subset expectation maximisation reconstruction. (c) Filtered back projection reconstruction method preceded by Butterworth filtering. (d) Ordered subset expectation maximisation reconstruction followed by Butterworth filtering. Intermediate count level. The images are individually scaled to their own maximum.

**Figure 4:**
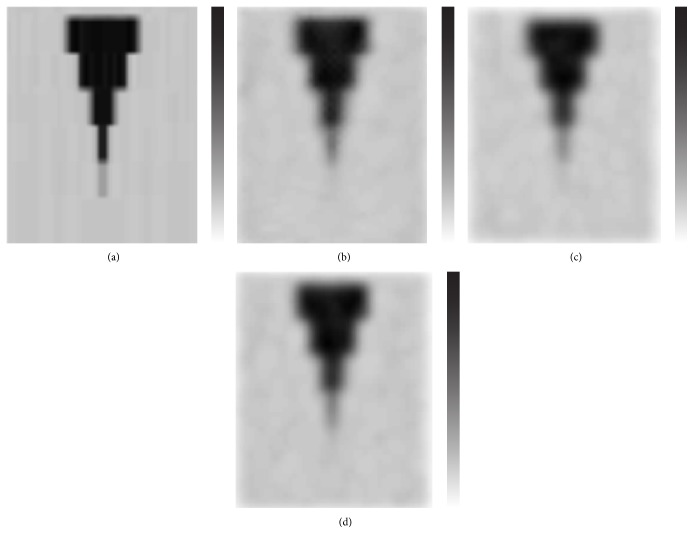
Reformatted coronal slices of the phantom. (a) Images reconstructed from noise-free projection images using ordered subset expectation maximisation reconstruction. (b) Autoregressive filtering before and after ordered subset expectation maximisation reconstruction. (c) Filtered back projection reconstruction method preceded by Butterworth. (d) Ordered subset expectation maximisation reconstruction followed by Butterworth filtering. Stacks with the highest activity. Intermediate count level. The images are individually scaled to their own maximum.

**Figure 5:**
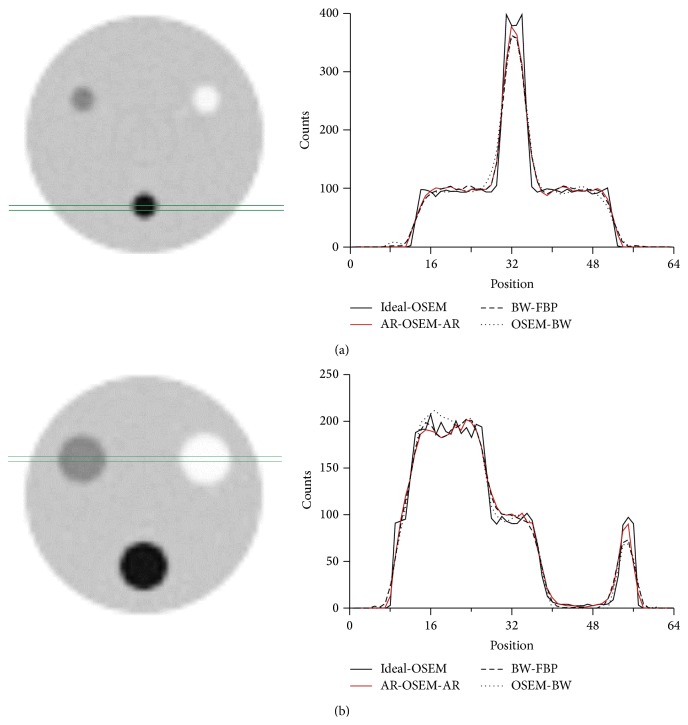
One-pixel-thick profiles drawn through the phantom. (a) Profiles at the level of a cylinder with a diameter of 20 mm. (b) Profiles at the level of cylinders with a diameter of 40 mm. Ideal OSEM: images reconstructed from noise-free projection images using ordered subset expectation maximisation reconstruction; AR-OSEM-AR: autoregressive filtering before and after ordered subset expectation maximisation reconstruction; BW-FBP: filtered back projection reconstruction method preceded by Butterworth filtering; OSEM-BW: ordered subset expectation maximisation reconstruction followed by Butterworth filtering. Intermediate count level. Profiles were rescaled so that they had the same amount of counts as the profile of the image reconstructed from noise-free projection images using ordered subset expectation maximisation reconstruction.

**Figure 6:**
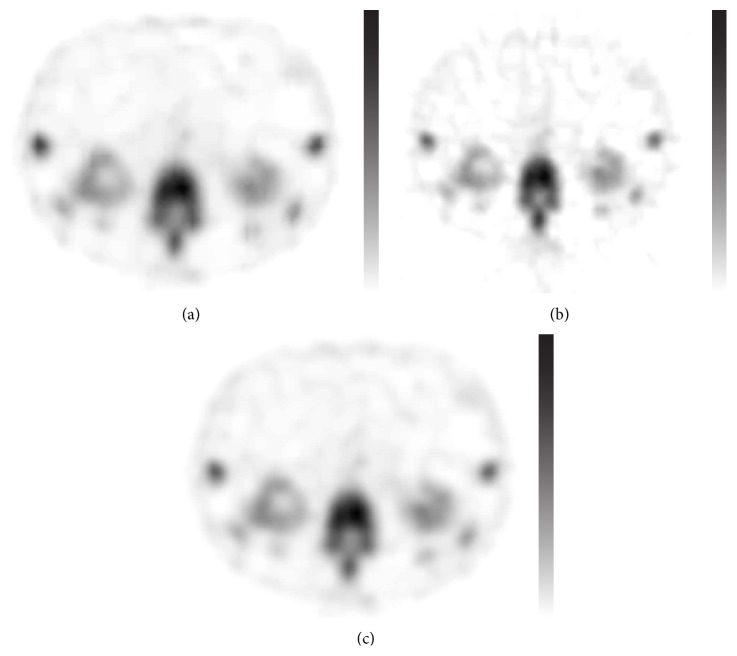
Transaxial slice of skeletal SPECT. (a) Autoregressive filtering before and after ordered subset expectation maximisation reconstruction. (b) Filtered back projection reconstruction method preceded by Butterworth filtering. (c) Ordered subset expectation maximisation reconstruction followed by Butterworth filtering.

Visually, the differences between the images produced by the three methods were small (Figures 3, 4, and 5). When comparing the skeletal SPECT data, the BW-FBP method showed lower image quality than the two other methods because of streak artefacts (Figure 6).

## 4. Discussion

This paper presented an improved two-dimensional adaptive AR filter and introduced a three-dimensional adaptive AR model for reduction of noise in SPECT images. We demonstrated that the quality of scintigraphic images can be improved when the same AR procedure is not applied to the original image and the summed error term image. We have previously shown that if a prediction region of four orthogonal neighbours of the predicted pixel with a block size of 5 × 5 pixels is used for both the original image and the summed error term image in the same simulation conditions, then the mean squared errors for the three different images with Poisson statistics are 0.85, 2.23, and 7.12 [4]; that is, this combination exhibits lower performance than any of those presented in Table 1.

The goal of filtering in SPECT is to suppress statistical noise and simultaneously preserve contrast and spatial resolution [1]. In the present study we showed that the AR-OSEM-AR method simultaneously provides both efficient noise rejection and good spatial resolution for hot objects. The methodological comparison was done using the well-known* de facto* reconstruction standards, FBP and OSEM, and by using one of the most commonly used filters in nuclear medicine, the Butterworth filter.

The BW-FBP method produced better performance than the two other methods in the analysis of the cold stacks. FBP's good performance with cold features has been noticed before [5, 13]. OSEM's built-in nonnegativity constraint explains its poor contrast in cold regions. In the analysis of the hot stacks, the magnitude of the differences between the three methods proved to be small, but the AR-OSEM-AR method had statistically the best performance. This is obviously due to the fact that part of the counts at the edges of the error term images could be returned back to the filtered image. Adding postfiltering to the method produced efficient noise reduction without compromising contrast or spatial resolution significantly.

The FBP method consists of filtering of the projection data and back projection of the filtered data [5, 10]. Prefiltering is generally not applied in the OSEM method because it lowers spatial resolution. Secondly, OSEM assumes that projection pixel values are independent and the number of counts is Poisson-distributed. Filtering might hamper these assumptions. OSEM reconstructed images are usually postfiltered because the images become noisier as the iterations proceed.

The disadvantage of FBP is that it can produce radial streak artefacts because filtered noisy projection profiles do not cancel each other out in back projection. In the present study, the phenomenon was seen in the clinical data. Iterative reconstruction algorithms also provide some other advantages over FBP. They permit the use of several important corrections, such as scatter, attenuation, and collimator response corrections, which can be included in the image reconstruction procedure. Incorporation of anatomical information derived from magnetic resonance imaging or computerized tomography is possible as well [14]. For the abovementioned reasons, FBP has in recent years been progressively replaced with iterative reconstruction algorithms.

The Butterworth filter is defined by two parameters: cut-off frequency and order [2]. In the present study, order was set to 2 because a ringing artefact is imperceptible to Butterworth filters of order 2 but can become a significant factor in filters of a higher order [15]. Filter orders much higher than 2 are often seen in clinical praxis. Edge sharpness in images produced by the BW-FBP and OSEM-BW methods can be improved by increasing the cut-off frequency, but the improvement occurs at the expense of increased noise.

In our opinion, a strength of the AR-OSEM-AR method is its simplicity, but the lack of user-controlled variables can also be regarded as a limitation. Sometimes, adjustable parameters are needed. No particular filter can emerge as the best filter for any organ system. However, filtering should be performed locally in the spatial domain, not globally in the frequency domain, because the correct trade-off between resolution and smoothing will vary at different points within the image.

Because the AR-OSEM-AR method was only marginally better than the OSEM-BW method, an additional study is needed to find out whether image quality will be even better if the AR method is applied to the intermediate results in between the iterations. Secondly, the AR-OSEM-AR method has not yet been tested with positron emission tomography (PET) data, but the method should also be suitable for PET data. The signal-to-noise ratio is considerably higher in PET than in SPECT. Therefore, our model will probably provide a good fit for PET data.

## 5. Conclusions

The AR-OSEM-AR method is a feasible denoising method in SPECT. It has good spatial resolution for hot features and it is simple to use. It does not have any adjustable parameters.

## Figures and Tables

**Table 1:** Effect of changing the block size of the summed error term image with a prediction region of 3 × 3 pixels. For the predictable image a prediction region of four orthogonal neighbours with a block size of 5 × 5 pixels was used.

Total counts	Block size	Mean squared error
28705	5 × 5	0.87
28705	6 × 6	0.86
28705	7 × 7	0.86

54469	5 × 5	2.12
54469	6 × 6	2.10
54469	7 × 7	2.14

108938	5 × 5	6.61
108938	6 × 6	6.56
108938	7 × 7	7.04

**Table 2:** Percentage coefficient of variation for different reconstruction techniques.

Method	Count level	RelAct	CF (cycles/cm)	CoV%
AR-OSEM-AR	50000	0	—	6.35
BW-FBP	50000	2	0.83	6.37
OSEM-BW	50000	4	0.84	6.36

AR-OSEM-AR	100000	0	—	4.60
BW-FBP	100000	2	0.80	4.56
OSEM-BW	100000	4	0.84	4.65

AR-OSEM-AR	150000	0	—	4.43
BW-FBP	150000	2	0.89	4.50
OSEM-BW	150000	4	0.86	4.43

AR-OSEM-AR: autoregressive filtering before and after ordered subset expectation maximisation algorithm; BW-FBP: Butterworth prefiltering and filtered back projection; OSEM-BW: ordered subset expectation maximisation algorithm and Butterworth postfiltering; RelAct: activity relative to background activity of 1; CF: cut-off frequency. The order of the filter was 2; —: not definable.

**Table 3:** Percentage contrast resolution values for the different methods.

Method	RelAct	10 Ø	20 Ø	40 Ø	60 Ø
A					
AR-OSEM-AR	0	18.4	56.3	74.6	84.2
BW-FBP	0	28.7	67.2	83.1	91.6
OSEM-BW	0	24.9	59.5	75.7	85.6

AR-OSEM-AR	2	9.0	86.1	84.3	103.5
BW-FBP	2	16.7	75.5	86.6	97.2
OSEM-BW	2	9.9	65.3	76.9	98.3

AR-OSEM-AR	4	136.5	253.0	270.4	296.5
BW-FBP	4	84.9	229.2	259.2	289.1
OSEM-BW	4	70.2	204.1	247.1	300.0

B					
AR-OSEM-AR	0	21.5	58.0	76.1	84.8
BW-FBP	0	20.4	61.6	83.2	91.8
OSEM-BW	0	32.9	61.9	78.0	86.6

AR-OSEM-AR	2	32.8	81.0	81.9	95.7
BW-FBP	2	35.1	80.2	87.4	102.7
OSEM-BW	2	29.7	75.4	78.8	98.3

AR-OSEM-AR	4	132.8	230.2	269.8	295.7
BW-FBP	4	94.6	236.9	270.3	316.2
OSEM-BW	4	85.6	224.6	255.1	308.5

C					
AR-OSEM-AR	0	24.0	55.9	75.0	85.5
BW-FBP	0	32.9	68.7	83.0	93.0
OSEM-BW	0	31.2	60.7	76.8	87.8

AR-OSEM-AR	2	41.1	76.0	86.9	94.9
BW-FBP	2	38.8	82.4	93.9	103.6
OSEM-BW	2	34.3	83.1	85.5	100.6

AR-OSEM-AR	4	119.4	237.1	266.3	297.7
BW-FBP	4	112.7	248.5	274.5	318.2
OSEM-BW	4	108.1	260.5	261.0	316.9

A: low count level; B: intermediate count level; C: high count level; AR-OSEM-AR: autoregressive filtering before and after ordered subset expectation maximisation algorithm; BW-FBP: Butterworth prefiltering and filtered back projection; OSEM-BW: ordered subset expectation maximisation algorithm and Butterworth postfiltering; RelAct: activity relative to background activity of 1; Ø: diameter.

**Table 4:** Full width at half maximum values for the different methods.

Method	RelAct	10 Ø	20 Ø	40 Ø	60 Ø
A					
AR-OSEM-AR	0	19.7	25.0	45.8	64.8
BW-FBP	0	17.9	23.2	43.5	63.8
OSEM-BW	0	19.4	23.2	43.6	62.8

AR-OSEM-AR	2	**—**	21.5	35.3	59.1
BW-FBP	2	**—**	23.2	36.6	59.6
OSEM-BW	2	**—**	22.2	35.8	59.1

AR-OSEM-AR	4	13.4	18.4	37.8	57.8
BW-FBP	4	15.8	20.4	37.9	57.9
OSEM-BW	4	15.2	19.9	37.7	57.9

B					
AR-OSEM-AR	0	23.7	23.7	43.0	62.3
BW-FBP	0	18.6	22.4	41.2	61.4
OSEM-BW	0	18.8	23.2	41.9	61.7

AR-OSEM-AR	2	19.4	19.8	38.9	59.0
BW-FBP	2	19.0	21.0	38.5	58.9
OSEM-BW	2	17.7	19.9	38.5	59.0

AR-OSEM-AR	4	13.0	19.7	37.8	58.2
BW-FBP	4	15.4	21.0	37.7	57.9
OSEM-BW	4	14.2	20.2	37.9	58.1

C					
AR-OSEM-AR	0	20.2	23.9	40.9	63.2
BW-FBP	0	18.5	21.1	40.6	61.7
OSEM-BW	0	16.8	23.2	40.3	62.3

AR-OSEM-AR	2	15.8	19.5	39.0	58.6
BW-FBP	2	16.9	20.8	38.6	58.7
OSEM-BW	2	16.5	20.3	39.1	58.6

AR-OSEM-AR	4	12.5	19.0	37.6	58.2
BW-FBP	4	14.0	19.6	36.8	58.0
OSEM-BW	4	13.8	19.3	37.4	57.9

A: low count level; B: intermediate count level; C: high count level; AR-OSEM-AR: autoregressive filtering before and after ordered subset expectation maximisation algorithm; BW-FBP: Butterworth prefiltering and filtered back projection; OSEM-BW: ordered subset expectation maximisation algorithm and Butterworth postfiltering; RelAct: activity relative to background activity of 1; Ø: diameter.
